# Qualitative and Quantitative Features of Music Reported to Support Peak Mystical Experiences during Psychedelic Therapy Sessions

**DOI:** 10.3389/fpsyg.2017.01238

**Published:** 2017-07-25

**Authors:** Frederick S. Barrett, Hollis Robbins, David Smooke, Jenine L. Brown, Roland R. Griffiths

**Affiliations:** ^1^Department of Psychiatry and Behavioral Sciences, School of Medicine, Johns Hopkins University Baltimore, MD, United States; ^2^Department of Humanities, Peabody Institute, Johns Hopkins University Baltimore, MD, United States; ^3^Center for Africana Studies, Krieger School of Arts and Sciences, Johns Hopkins University Baltimore, MD, United States; ^4^Department of Music Theory, Peabody Institute, Johns Hopkins University Baltimore, MD, United States; ^5^Department of Neuroscience, School of Medicine, Johns Hopkins University Baltimore, MD, United States

**Keywords:** psilocybin, music information retrieval, music theory, music perception

## Abstract

Psilocybin is a classic (serotonergic) hallucinogen (“psychedelic” drug) that may occasion mystical experiences (characterized by a profound feeling of oneness or unity) during acute effects. Such experiences may have therapeutic value. Research and clinical applications of psychedelics usually include music listening during acute drug effects, based on the expectation that music will provide psychological support during the acute effects of psychedelic drugs, and may even facilitate the occurrence of mystical experiences. However, the features of music chosen to support the different phases of drug effects are not well-specified. As a result, there is currently neither real guidance for the selection of music nor standardization of the music used to support clinical trials with psychedelic drugs across various research groups or therapists. A description of the features of music found to be supportive of mystical experience will allow for the standardization and optimization of the delivery of psychedelic drugs in both research trials and therapeutic contexts. To this end, we conducted an anonymous survey of individuals with extensive experience administering psilocybin or psilocybin-containing mushrooms under research or therapeutic conditions, in order to identify the features of commonly used musical selections that have been found by therapists and research staff to be supportive of mystical experiences within a psilocybin session. Ten respondents yielded 24 unique recommendations of musical stimuli supportive of peak effects with psilocybin, and 24 unique recommendations of musical stimuli supportive of the period leading up to a peak experience. Qualitative analysis (expert rating of musical and music-theoretic features of the recommended stimuli) and quantitative analysis (using signal processing and music-information retrieval methods) of 22 of these stimuli yielded a description of peak period music that was characterized by regular, predictable, formulaic phrase structure and orchestration, a feeling of continuous movement and forward motion that slowly builds over time, and lower perceptual brightness when compared to pre peak music. These results provide a description of music that may be optimally supportive of peak psychedelic experiences. This description can be used to guide the selection and composition of music for future psychedelic research and therapy sessions.

## Introduction

Psychedelic drugs (i.e., classic hallucinogens, or serotonin 2A receptor agonists) have profound effects on perception, cognition, and states of consciousness (Nichols, 2016; Preller and Vollenweider, 2016). Early research in the 1950s and 1960s on psychedelics investigated the therapeutic value of these drugs (Garcia-Romeu et al., 2016). There has been a recent resurgence of this line of therapeutic research, with reports demonstrating potential therapeutic benefits of psychedelics for mood disorders and addiction (Grob et al., 2011; Johnson et al., 2014; Bogenschutz et al., 2015; Carhart-Harris et al., 2016; Griffiths et al., 2016; Ross et al., 2016). A unique effect of hallucinogens is that they may occasion mystical or non-dual experiences (Barrett and Griffiths, 2017), and these experiences may hold therapeutic value (Garcia-Romeu et al., 2015; Griffiths et al., 2016; Ross et al., 2016). Non-dual experiences involve a relaxation of the typical subject-object (self vs. other, intenal vs. external, good vs. bad, us vs. them, etc) perspective that pervades typical waking consciousness (Josipovic, 2014). Mystical experiences are described as non-dual experiences that include a deeply felt positive mood, difficulty putting the experience into words, and an experience where traditional notions of time and space do not have meaning (Stace, 1960), and they are operationally defined and investigated in psychedelic research through the use of instruments such as the Mystical Experience Questionnaire (MacLean et al., 2012; Barrett et al., 2015).

While psychedelics, including psilocybin, may hold promise as future therapeutics for some types of intractable disorders (such as addiction and treatment-resistant depression), there is significant variability in response to these drugs. The success of psychedelics as therapeutics may rely on the optimization of the delivery of these drugs, including the therapeutic context within which they are administered. Optimization of therapeutic context, including the music that is presented during psychedelic therapy, may be crucial to the conduct of a successful therapy session.

Music listening can sometimes lead to altered states of consciousness, including trance (Rouget, 1985), absorption (Sandstrom and Russo, 2013), groove (Janata et al., 2012), states that are similar to flow states (Csíkszentmihályi, 1990), and states of religious ecstasy (Penman and Becker, 2009). It is believed that music listening can provide psychological support during the acute effects of psychedelic drugs, and may even support the occurrence of mystical or non-dual experiences (Eisner and Cohen, 1958; Eisner, 1997). Consequently, music listening has been incorporated into research (Johnson et al., 2008) and clinical (Bonny and Pahnke, 1972; Richards, 2015) psychedelic drug sessions during acute drug effects.

Psychedelics have recently been shown to alter emotional experience during music listening, specifically increasing the experience of positive emotions including transcendence (Kaelen et al., 2015). Music listening during psychedelic experiences may also lead to increased entropy in brain activity, which may be a mechanism by which long-term changes in personality and behavior are realized (Lebedev et al., 2016). This entropic change may be a necessary process for realizing transformative experience during psychedelic sessions, including mystical or non-dual experiences or experiences involving altered sense of self (Carhart-Harris et al., 2014).

Music has been used for many decades in psychedelic research and therapy sessions (Eisner and Cohen, 1958; Bonny and Pahnke, 1972; Richards, 2015). Early research into the therapeutic value of psychedelics provided the basis for recommendations of specific musical selections that were believed to complement the psychedelic experience by providing continuity, structuring the experience, narrowing attention, heightening concentration, and releasing emotion (Bonny and Pahnke, 1972). These musical selections were primarily identified using the intuition of a therapist, and many playlists currently being used by research sites around the world have been developed based on older playlists and/or the intuition of more recent therapists. While early reports (Bonny and Pahnke, 1972) suggested that there were characteristics of music (such as instrumentation, forward movement, phrasing and dynamics, melodic line, stability of rhythm, and overall subjective mood) that were critical for supportive music during different phases of the psychedelic experience (e.g., phases of pre peak experience, peak experience, and post-peak experience), descriptions of these phases were unfortunately vague and the characteristics of the music that was said to be supportive during different phases of psychedelic experience was often similar if not identical between phases. Thus, while influential, recommendations made by Bonny & Pahnke are non-specific and limited. The process of selecting music for psychedelic therapy sessions has not yet been clearly defined, and features of music thought to be supportive during the different phases of drug effects have not been well characterized, empirically derived, or empirically tested.

## The current study

Identifying and characterizing features of music that is already being used to support peak experiences with psychedelics is a logical first step toward addressing the open question of whether particular musical characteristics are optimal. Accordingly, we created an online survey within which therapists with expertise in the use of music in psilocybin therapy sessions were asked to identify pieces of music that they felt were optimally supportive during mystical or non-dual peak experiences, as well as during the period leading up to peak experience (i.e., “pre peak” music) after ingesting psilocybin or psilocybin-containing mushrooms. The survey was restricted to consideration of psilocybin in therapy in order to avoid any potential confounding effects of the pharmacological differences between psychedelics (e.g., psilocybin does not have affinity for dopamine receptors, whereas LSD does). The survey was restricted to therapists, rather than those receiving psilocybin for any given purpose, since individual responses to music can be idiosyncratic with both the patient and the goals of a psilocybin session (e.g., personal growth, exploration of consciousness, or seeking healing), and therapists are more likely to have a generalizable sense of what types of music may be optimally supportive for individuals undergoing psilocybin therapy or research procedures.

Research drawn from the disciplines of psychology and music education can offer qualitative and quantitative methods to characterize music that is supportive during a psychedelic experience. Qualitative methods include subjective ratings of descriptive features of music such as compositional form, orchestration, phrase structure, tempo, mode, tonal stability, articulation, dynamics, and meter. These are classic dimensions of music derived from music theory and compositional pedagogy that can be used to classify and categorize periods of music, styles of music composition, and elements of performance. Qualitative ratings have been used in past research, for example, to identify aspects of music that define the affective and expressive value of music (Hevner, 1935, 1936, 1937). Qualitative and observational data have also been used to guide the composition of novel music. For example, observational data has been used in film scoring for nearly a century (Wierzbicki, 2009). Furthermore, observational data has been used to inform composition exercises in music instruction for centuries, beginning with seminal music composition pedagogues such as Johann Fux's composition treatise from 1725 (Fux et al., 1965), Carl Czerny's composition treatise (Czerny and Bishop, 1848), and continuing through today (e.g., Eckert, 2005). Prominent collegiate music theory textbooks also include composition exercises where students are asked to write novel music given a list of required elements (e.g., Clendinning and Marvin, 2005; Laitz, 2012). Thus, qualitative ratings made by qualified experts in music theory and/or music cognition can be conducted on recordings of musical performances, and constitute a human observational method that can yield the type of information that could be used to compose novel music of a similar type.

Quantitative methods for characterizing music include a range of approaches employed in the field of Music Information Retrieval (MIR) (Tzanetakis and Cook, 2002). MIR methods employ signal processing and other computational algorithms to analyze the raw audio signal from a recorded piece of music. Such methods have been utilized in a wide range of applications, including automated music recommendation systems (Schedl, 2017), semantic music analysis systems (Herremans and Chuan, 2017), and automated systems for the composition of novel music (Cope, 2000, 2005; Lucas et al., 2016; Williams et al., 2017).

A strength of MIR methods is that they can yield objective measures of physical properties of sound that align with subjective, perceptual dimensions of music listening experience (Alluri and Toiviainen, 2010) and that may relate to subjective experiences such as emotions that accompany music listening (Eerola et al., 2012). Perceptual dimensions of music listening are derived from adjective ratings of the qualities of sounds that are often arranged in bi-polar scales such as dullness-sharpness, warm-cold, colorless-colorful, and soft-hard. Brightness as a perceptual dimension can be considered in terms of the comparison between a sense of dull-sharp, dark-bright, and colorless-colorful, with greater “sharp,” “bright,” and “colorful” nature of a sound relating to greater overall perceptual “brightness” of that sound. Activity can be considered in terms of the comparison between soft-hard, weak-strong, and low energy-high energy, with greater “hard,” “strong,” and “high-energy” relating to greater “activity.” Fullness can be considered in terms of the comparison of empty-full and scattered-compact, with greater “full” and “compact” relating to greater “fullness.” Computational measures that relate to perceptual dimensions of brightness, fullness, and activity can be derived from raw audio signal for individual musical selections. These measures constitute objective information that can both be used to characterize musical selections, and compose novel music of a similar type.

The musical pieces identified by therapists in the current survey underwent qualitative analysis by two experts in music theory and music cognition, who identified the musical features (e.g., compositional form, phrase structure) common among the indicated musical selections for each period of drug effects (“peak music” and “pre peak music”). These pieces were also submitted to quantitative analysis using music information retrieval (MIR) methods to provide complementary quantitative measures of features that were common among peak music pieces and among pre peak music pieces, and that have been linked to both perceptual correlates of music listening (Alluri and Toiviainen, 2010) and brain activity during music listening (Alluri et al., 2012). From these methods, we propose an initial list of features of music that may be optimally supportive during peak psychedelic experiences, though the generalizability of this list of features to experiences with psychedelics other than psilocybin remains to be established.

## Methods

We conducted an online survey within which we asked expert psilocybin guides (those who have guided more than 50 individuals in psilocybin therapy sessions) to identify two to three pieces of music that they have found to be optimally supportive of mystical or non-dual peak experiences during the acute effects of psilocybin and/or psilocybin-containing mushrooms for most individuals (“peak music”). We also asked guides to indicate two to three pieces of music found to be supportive of mystical or non-dual peak experiences, that would be played during the period leading up to a mystical or non-dual experience (“pre peak music”). Finally, we asked guides to provide details regarding the characteristics that typically define the therapeutic sessions with psilocybin that they conduct.

### Participants

Participants were recruited by word of mouth recommendation to complete an anonymous survey of the role of music in supporting psychedelic therapy sessions. Participants were invited to complete the survey if they were at least 18 years old, could read, write, and speak English fluently, and had experience conducting or guiding therapeutic sessions with a high dose of the classic hallucinogen psilocybin (or psilocybin-containing mushrooms) that included music listening during the session. We did not restrict survey participation to those guides who were completing legally sanctioned psilocybin therapy sessions, and thus, we felt that anonymous participation was necessary. The aims of the study and basic information about study completion were provided at the beginning of the survey. Participants were informed that study participation was anonymous, they could choose to stop answering questions at any time, and if they did not complete the survey, their specific responses would not be used. Participants were also informed that their completion of this survey would serve as their consent to be in this research study. For a participant's data to be retained, they had to complete the survey and indicate at the end of the survey that they provided both complete information and consent for us to use their responses. In order to restrict analyses to responses from the therapists with extensive experience, participants' data were included if they indicated having guided at least 50 different individuals through a session with psilocybin or psilocybin-containing mushrooms. Written informed consent was not obtained from participants since this was an anonymous internet survey. All procedures were approved by the Institutional Review Board of the Johns Hopkins University School of Medicine.

### Materials

#### Demographic questionnaire

Participants were asked to provide minimal demographic information, consisting of gender and age. Age response options consisted of the following: 18–24, 25–34, 35–44, 45–54, 55–64, 65–74, and 75+.

#### Survey of psychedelic therapy experience

Participants were asked to provide details regarding their experience guiding sessions with psilocybin. Participants were asked to indicate the approximate number of high-dose psilocybin sessions they had guided, the approximate number of different people they had guided in psilocybin sessions, the approximate number of group sessions (i.e., guiding two or more people simultaneously) they had guided, and approximately how many individual psilocybin sessions (i.e., guiding one person) they had guided. Participants were then asked to complete a series of questions that characterize the conditions of a typical high-dose psilocybin session: typical body posture of volunteers (lying down, sitting up, or standing/walking), whether the individual receiving psilocybin typically wore eyeshades (yes/no), the typical location of high-dose sessions (inside/outside), whether a co-guide was typically present (yes/no), the percentage of sessions that included music, the percentage of time during a typical session that included music, and the typical goals for a high-dose psilocybin session (healing, spiritual growth, insight, non-dual/mystical experience, or other).

#### Identification of pre peak and peak music

Participants were asked to provide the title, artist, and album of “two to three pieces of music that you feel are optimally supportive of mystical or non-dual peak experiences during the acute effects of psilocybin for most individuals” and “two to three pieces of music that you feel are optimally supportive of mystical or non-dual peak experiences, that would be played during the period leading up to a mystical or non-dual experience.” Mystical or non-dual experiences were defined as “being characterized by a profound feeling of oneness or unity with the surroundings, with the world, with the universe, or with all that exists,” consistent with previous descriptions of mystical experience (James, 1902; Stace, 1960).

### Qualitative analysis

Three authors, experts in music theory (DS, JLB) and music cognition (JLB, FSB), conducted an initial review of peak and pre peak music provided by survey participants. The three authors then compared notes and used consensus to develop an initial list of qualitative musical and acoustic features for subsequent rating of peak and pre peak music provided by study participants. Two authors (DS and JLB) subsequently listened to peak and pre peak musical selections indicated by survey participants, and rated all consensus features for each musical selection. During listening, the feature list was refined and reduced to those features that were prominently expressed in either peak or pre peak musical selections. Qualitative ratings were discussed, and the final list of features that were shared within but differed between peak or pre peak musical selections (listed in Table 1) was confirmed by consensus discussion among three authors (DS, JLB, FSB).

**Table 1:** Qualitative features of pre peak and peak music.

**Musical feature**	**Pre peak music**	**Peak music**
Compositional form	○ Less likely to be cyclical or contain a slow build than peak music; there may be multiple sections throughout the composition	○ Cyclical or slow build to climax
Phrase structure	○ Either rather static or very variant (sectionalized, many ups and downs) ○ Long duration between phrases, much longer than “breath length” ○ Few or no discernible melodies ■ Rare melodies were short ■ Long duration between melodies ■ The instrument that carried the melody was often changing ○ Few accented events ○ Some startling openings ○ Meandering ○ Many different sections ○ Overall, less unified than peak music	○ Regular, consistent, formulaic ○ Either: ■ Moderate to long duration (4 or 8 bar phrases) ● Setting up expectations that would be met at regular intervals ● Likely to be cyclical ● Endlessly spinning phrases ■ Breath-length phrases ● Irregular, uneven length ● Long length ● Typically in unmetered pieces ○ Instrument carrying the melody was consistent
Dynamics	○ Typically steady; unchanging within sections	○ Static or slowly building ○ Few sudden events
Meter	○ Simple, mostly quadruple meter ○ No mixed meter	○ Simple, mostly quadruple meter (sparse triple) ○ Mixed meter rare ○ Metric pulse: clear in some, absent in others ○ Fewer instruments agreed on the pulse at the same time than in pre peak music ○ Rubato was uncommon
Tempo	○ Melody notes change at a slightly faster pace than peak music	○ Notes change around 60 BPM or slower; melodies seem restful and unrushed
Orchestration	○ Heterogeneous instrumentation (changed more often than peak music) ○ Instruments more recognizable than peak music ○ Some drone ○ Drumming for forward motion ○ Vocals present, but few lyrics	○ Homogenous instrumentation (stayed the same within a piece of music) ○ Instruments designed to be “unrecognizable” ■ Tonic drone (often prolonged by an instrument such as a tanbura or a didgeridoo) ■ Non-western instruments (bamboo flute, didgeridoo) ■ Vocals unintelligible or in an obscure language ■ Overtone singing ■ Ensemble, such as strings (no one instrument can typically be identified) ■ 80 s synth-pad sounds ■ Brass instruments uncommon ○ World-music influence ○ Drumming, but not for forward motion (as in a one measure drum loop that is repeated without much change) Use of cyclic, tabla drumming more common than drumset ○ Strings more common than in pre peak music ○ Reverb was used often ○ Pieces often began with one or two tracks (such as a drone or drumming). More instruments were slowly added and lasted for the entirety of the composition
Mood/Tone	○ Darker than peak music ○ More frequent changes of mood than peak music	○ Few changes of mood throughout the composition
Mode	○ The minor mode is possible, more so than in peak music. A lowered ^∧^7 is more common than a raised leading tone ○ mode is more variable than peak music	○ Mostly major
Articulation		○ More legato than pre peak music
Tonality		○ Triadic harmonies ○ Typical chord progressions ○ Tonic drone common ○ Few modulations (if any)
Genre	○ Either: ■ New age (potentially with a world music influence) ■ Classical (often orchestra and chorus)	○ Either: ■ New age (potentially with a world music influence) ■ Classical (often orchestra and chorus)
General observations	Often, what happens in first minute or two of a piece will have no relation to what happens later in the piece. There is less of a sense of large-scale directed motion	Melodic material, phrase length, harmonies, mode, or whatever you set up at the beginning of the piece will more or less be constant throughout the piece

### Quantitative analysis

Subjective perceptual features of music, such as brightness, fullness, and activity, can be identified and consistently coded by non-expert musical raters (Alluri and Toiviainen, 2010). These subjective perceptual features of music have been associated, using correlation, regression, and principal component analysis methods (Alluri and Toiviainen, 2010; Alluri et al., 2012, 2013) with a set of stimulus features that are computationally derived using music information retrieval (MIR) methods (Tzanetakis and Cook, 2002). The MIR features with which brightness, fullness, and activity have been associated include timbral features (zero-crossing rate, ratio of high to low energy, spectral centroid, spectral entropy, spectral rolloff, spectral flatness, total spectral flux, sub-band spectral flux, and roughness), tonal features (key, key clarity, and mode), temporal features (pulse clarity, event density), and root-mean-squared (RMS) energy. We calculated these MIR features for each musical selection and used principal components analysis to derive a factor score (and the primary quantitative outcome measures) for brightness, fullness, and activity for each musical stimulus, as previously described (Alluri and Toiviainen, 2010; Alluri et al., 2012, 2013).

Key, key clarity, and mode were calculated as timecourses with a frame size of 1 s and 50% overlap in frames (Lartillot and Toiviainen, 2007). Pulse clarity was calculated as a timecourse with a frame size of 5 s and 10% overlap in frames, and event density was calculated as a timecourse with a frame size of 10 s and no overlap in frames. All other features were calculated as time courses with a frame size of 50 ms and a 50% overlap in frames. The values that were analyzed for each MIR feature were the mean of the time courses of these features for the entire musical selection (as in Alluri and Toiviainen, 2010). MIR features were extracted using the Music Information Retrieval Toolbox (Lartillot and Toiviainen, 2007).

The average value of each MIR feature from both peak and pre peak music was entered into principal components analysis with varimax rotation to identify combinations of MIR features that represent previously proposed music-perceptual dimensions of Activity, Brightness, and Fullness in acoustic features (Alluri and Toiviainen, 2010; Alluri et al., 2012). The first 9 principal components were extracted as in previous reports (Alluri et al., 2012), and explained 94% of the variance in the data. Principal components that identified brightness, fullness, and activity were identified by visual inspection of the MIR features that load onto each component, and matched to components with similar loading patterns that were previously described (Alluri et al., 2012). Component scores for each principal component (including brightness, fullness, and activity) were then compared between peak and pre peak stimuli using Welch's two-sample *t*-tests.

## Results

Thirteen individuals responded to the survey. Two individuals failed to complete the survey, and thus their responses were discarded. Indicated ages of the remaining 11 participants were 25–34, 55–64, 65–74 and 75+ in 1, 6, 2, and 1 participants, respectively. One participant declined to identify their age. One individual who completed the survey indicated that he or she had guided fewer than 50 individuals in psilocybin sessions and thus his or her responses were not included in analysis. This yielded a final sample of 10 participants (three females). All completed the survey and provided consent to use their data. Participants reported guiding a substantial number of individual (i.e., guiding a single volunteer) high-dose psilocybin sessions (M = 365 sessions, range = 60 to 2,000) in moderately large number of unique individuals (M = 213 different people, range = 50 to 1,200). Seven participants also indicated having guided group psilocybin sessions (M = 106 group sessions, range = 5 to 400 group sessions).

### Typical psilocybin session conditions

Six participants indicated that music was included at some point within 100% of the high-dose psilocybin sessions that they guided, while three indicated that music was present at some point in 90% or more of the total number of high-dose psilocybin sessions that they guided, and one participant indicated that music was present at some point in ~70% of the total number of high-dose psilocybin sessions that they guided. Five participants indicated that music was present during the entire session during a typical psilocybin session. One participant indicated music was present during 95% of the typical high-dose psilocybin session, three participants indicated that music was present during at least 80% of the typical high-dose psilocybin session, and one participant indicated that music was present during more than 70% of the typical high-dose psilocybin session.

All participants indicated that individuals receiving psilocybin or psilocybin mushrooms (volunteers) are typically lying down during psilocybin sessions, while three participants indicated that volunteers may also be sitting up at times during psilocybin sessions, and one participant indicated that volunteers may also be standing or walking at times. All participants indicated that volunteers are typically inside rather than outside during psilocybin sessions, while four participants also indicated that volunteers may be outside at times during psilocybin sessions. Nine participants indicated that volunteers typically wear eyeshades during psilocybin sessions, and seven participants indicated that they typically have a co-guide or assistant present for most of the sessions. All participants indicated that healing, insight, spiritual growth, and mystical or non-dual experience are all typical goals of volunteers during high-dose psilocybin sessions.

### Musical recommendations

Twenty-seven unique recommendations were made for peak music, and 27 unique recommendations were made for pre peak music. Three of these recommendations (*Adagio for Strings* by Samuel Barber, Symphony No. 3 by Henryk Górecki, and “Kyrie” from Mass in B Minor by J. S. Bach) were identified for both peak and pre peak stimuli, and were excluded from analysis since they were not specific to either peak or pre peak periods. Recordings for two recommendations for the peak period (*Sounds of the Soul #1* and *Sounds of the Soul #2* by Sheila Z. Stering & Gary Stadler, and “Awakening” from *Disciple* by Mark Seelig) and two recommendations for the pre peak period (*Ayni* by Tito la Rosa, and a track identified with title, artist, and album as “Angel Love”) were not available to the study team at the time that the analysis was conducted, and thus these stimuli were not included in the final analysis. This yielded 22 peak (Table 2) and 22 pre peak (Table 3) stimuli for final analysis.

**Table 2:** Stimuli recommended for peak music.

**Title**	**Artist**	**Album**
Mass in D; Missa Solemnis	Beethoven	Berliner Philharmonika
Prayer for Compassion—whole CD	David Darling	Prayer for Compassion—whole CD
Track 1. Shh/peaceful and Track 2. In a Silent Way	Miles Davis	In a Silent Way
Healing Chant	Dalai Lama	Healing Chant
Joy of Life	Ariel Kalma	Serenity
Ngai gamelan—whole CD	David Parsons	Ngai gamelan—whole CD
Temple of Silence	Deuter	Garden of the Gods
Girl/Boy Song	Aphex Twin	Richard D. James album
Wacah Chan/Cintamani	Loren Nerell/Mark Seelig	Tree Of Life
n.s.	Mickey Hart	Planet drum
Novus Magnificat	Constance Demby	Novus Magnificat
Journey of the Whales	Donnelan, Bocci and Robe	Vanishing Voices
Ur	David Byrne	Forest
Live in New York—Whole CD	G S Sachdev	Live in New York—Whole CD
Om Namah Shivaya	Russill Paul	the Yoga of Sound
Echoes	Pink Floyd	Meddle
Returning	Jennifer Berezen	Returning
Devi	Chloe Goodchild	Devi
All Our Ancestors	Tuu	Mesh
Ani Hu	Robert Jameson	Empathy with God
Darkwood IV	David Darling	Dark Wood
Call Of The Divine	Mark Seelig	Disciple
^*^Sounds of the Soul #1 and #2	Sheila Z. Sterling and Gary Stadler	Sounds of the Soul
^*^Awakening	Mark Seelig	Disciple
^†^Adagio for Strings	Samuel Barber	
^†^Symphony No. 3	Henry Gorecki	
^†^Kyrie	J.S. Bach	Mass in B Minor

*Entries are presented verbatim as provided by respondents to the survey. Cells that include “n.s.” indicate values that were not specified by the respondent*.

* *Recordings of these stimuli were unavailable at the time of analysis, and thus were excluded from analysis*.

† *Stimuli were identified for both peak and pre peak music, and were excluded from analysis since they were not specific to either peak or pre peak periods*.

**Table 3:** Stimuli recommended for pre peak music.

**Title**	**Artist**	**Album**
An eagle in your mind	Boards of Canada	Music has the right to children
Encounter	Byron Metcalf, Mark Seelig	Intention
Clair de Lune	Claude Debussy	n.s.
n.s.	Deva Preval	n.s.
Gnossiene 3	Erik Satie	n.s.
n.s.	Jaya Laksmi	Various
Golden Bowls	Karma Moffett	Golden Bowls
O Magnum Mysterium	Morten Lauridsen	Angels on High: Robert Shaw; Telarc 20 CD-80-461
Compassion	Peter Kater	Compassion
Whippoorwill	R. Carlos Nakai	Changes
Wachuma's Wave	Seve Roach, Byron Metcalf, Mark Seelig	Wachuma's Wave
Various	Snatam Khar	Different albums
Nightbloom Track One	Steve Roach, Mark Seelig	Nightbloom
Flying and Flocking	Zoe Keating	Into the Trees
Several songs including Violeta, Words of Truth	Stephen Micus	The Garden of Mirrors
Nimrod	Edward Elgar	Enigma Variations; Bernstein Artist's Album DGG 457 691-2
Stillpoint in Motion	Tuu	All Our Ancestors
German Requiem I and 2	Johannes Brahms	San Francisco Symphony/Blomstedt, London 443 771-2.
Alternesia	John Iverson	Alternesia
Comala	Jorge Reyes	Comala
Cloak of darkness	Phil Thornton	Cloak of darkness
Monsoon Point	Al Gromer Khan	Monsoon Point
^*^Angel Love	Angel Love	Angel Love
^*^Ayni	Tito la Rosa	Ayni
^†^Adagio for Strings	Samuel Barber	
^†^Symphony No. 3	Henry Gorecki	
^†^Kyrie	J.S. Bach	Mass in B Minor

*Entries are presented verbatim as provided by respondents to the survey. Cells that include “n.s.” indicate values that were not specified by the respondent*.

* *Recordings of these stimuli were unavailable at the time of analysis, and thus were excluded from analysis*.

† *Stimuli were identified for both peak and pre peak music, and were excluded from analysis since they were not specific to either peak or pre peak periods*.

### Qualitative analysis

Table 1 describes features of music that were rated by experts in music theory and music cognition (DS and JLB) to differentiate peak and pre peak music. The overall characteristics that define peak period music are the presence of regular, often breath-length phrase structure, in major mode or based on a single overtone series, simple meter, and steady metric pulse. Melodic material, phrase length, harmonies, instrumentation, and tonality of peak music will typically remain constant throughout a given piece, with few sudden events. Many musical pieces recommended for peak period are ensemble pieces utilizing instrumentation that makes it difficult to identify individual instruments or performers within the ensemble. Where specific individual instruments are featured, they tend to be from non-European cultures. In general peak period music is characterized by consistency, regularity, and either some sense of directed motion or a cyclical form.

With regard to pre peak music, there is typically less consistency within and among musical selections. For example, there were no qualitative musical or acoustic features that stood out as common among the majority of pre peak musical selections. Within a given piece of music, there might be little relationship between what happens in first minute or two of a piece and what happens later in the piece. There is also less of a sense of large-scale directed motion in pre peak music than in peak music.

### Quantitative analysis

**Figure 1:**
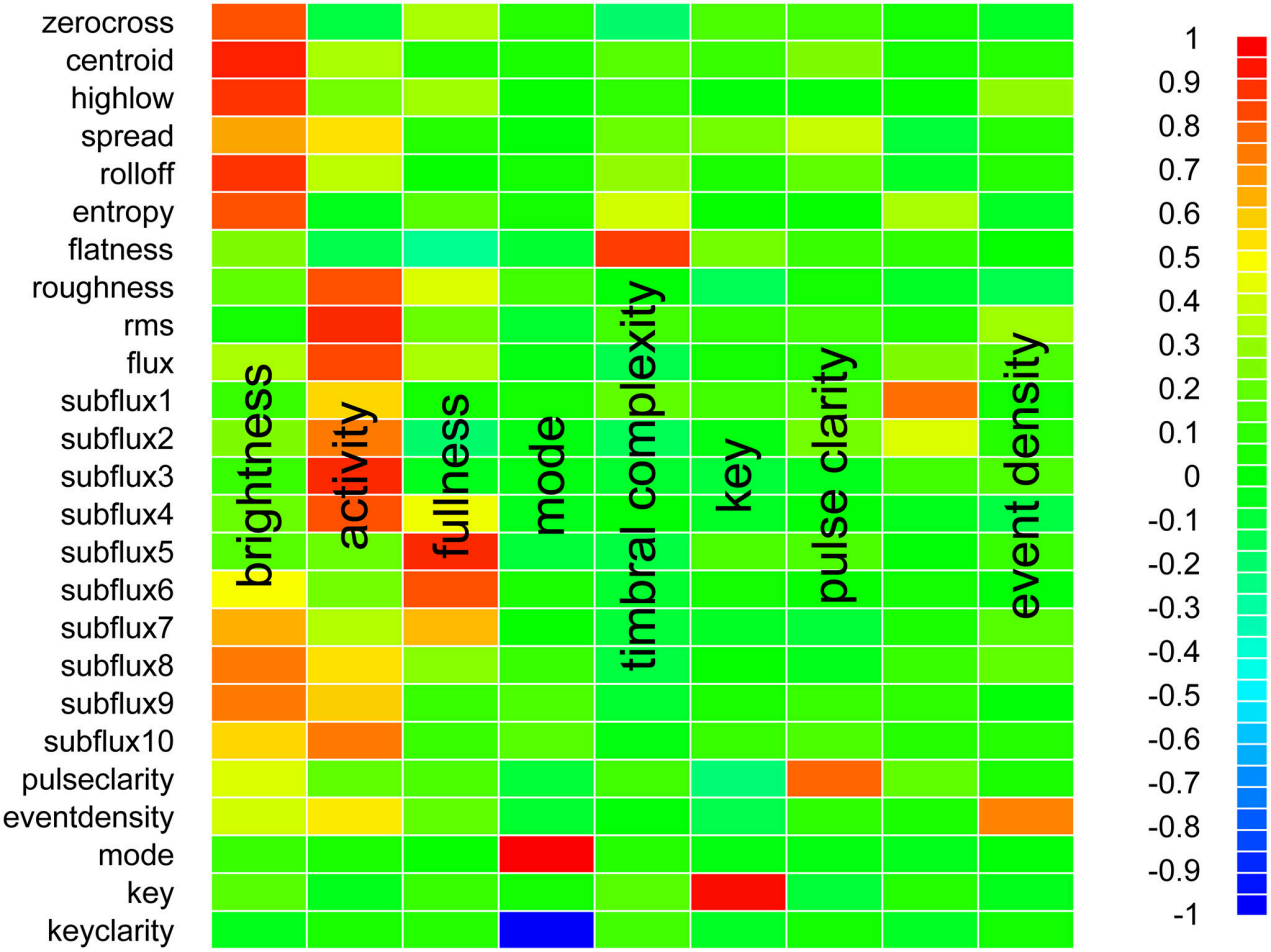
Principal component loadings of the music information retrieval features of musical stimuli. Music information retrieval (MIR) features derived from all musical stimuli (both peak and pre peak musical recommendations) were submitted to principal components analysis. Each row in the figure represents an MIR feature that was calculated for musical stimuli. Each column in the figure represents a component extracted from the MIR features of musical stimuli using principal component analysis. The shading of each cell indicates the loading of a given MIR feature on the respective principal component. The color bar on the right-hand side of the figure indicates the positive (red) or negative (blue) loading of each MIR feature on each principal component. Subjective labels for each principal component are overlaid on the figure. One of the components (represented in the 8th or penultimate column of the figure) did not map onto a previously identified perceptual dimension, and thus remains unlabeled.

Figure 1 describes the loadings of each MIR feature onto the first nine principal components. “Brightness” (component 1), “activity” (component 2), and “fullness” (component 3) dimensions were identified by visual inspection of the quantitative features that load onto each component, and matched to components with similar loading patterns that were previously described (Alluri et al., 2012). Individual dimensions for mode, key, pulse clarity, and event density were also identified in a similar fashion. Though the MIR feature *flatness* was previously identified in a component named “Timbral Complexity” (Alluri et al., 2012), and *flatness* was identified as primarily loading onto component 5 within the current data, the other features that were previously shown to load onto timbral complexity (*spectral spread* and *spectral centroid*) did not load strongly onto component 5. As with previous applications of this method (Alluri et al., 2012), one of the principal components (i.e., the 8th column within Figure 1) did not map clearly onto a previously identified perceptual dimension, and thus remains unlabeled.

Welch's two-sample *t*-tests indicated that estimated component scores for component 1 (Brightness) were greater for pre peak music (M = 0.19, SE = 0.12) than for peak music (M = −0.20, SE = 0.08, *t* = 2.636, *df* = 30.963 *p* = 0.013). Component scores for component 2 (Activity) were not significantly different between peak (M = −0.06, SE = 0.12) and pre peak music (M = −0.06, SE = 0.2, *t* = 0.028, *df* = 30.75, *p* = 0.978). Component scores for component 3 (Fullness) were numerically, but not significantly, greater for peak music (M = 0.21, SE = 0.21) than for pre peak music (M = −0.31, SE = 0.23, *t* = 1.66, *df* = 34.48, *p* = 0.106). No other component scores, nor any individual MIR features, were significantly different between peak and pre peak music.

## Discussion

Clear differences, especially in qualitative ratings, have been identified between music that was recommended for the peak period of psilocybin experience and music that was recommended for the period leading up to the peak period of psychedelic experience (the “pre peak” period). More importantly, clear similarities were found among stimuli that were recommended for the peak period of psilocybin effects, while there were few consistencies among stimuli that were recommended for the pre peak period.

### Qualitative analysis of musical selections

Peak period stimuli have a regular, predictable, formulaic phrase structure, and consistent instrumentation, with a characteristic feeling of continuous movement that may slowly build over a given piece of music. Peak period stimuli are also typically composed in a simple, often quadruple, meter, with a relatively slow tempo and homogenous instrumentation. For peak period musical selections in which specific individual instruments are featured, they tend to be from non-European cultures, which may presumably appear exotic to subjects undergoing the experience. Music with such qualities makes intuitive sense for the purposes of supporting an experience of unity, or where a felt sense of self is diminished or absent. Great variation during this time period, the introduction of jarring transitions, and lack of predictability in composition may lead to a sense of uneasiness, an attempt to predict what will happen next, or some form of vigilance or attention that might be disruptive to a state of consciousness marked by mystical experience.

### Quantitative analysis of musical selections

Peak period stimuli were found to have relatively lower perceptual brightness, and potentially greater perceptual fullness, than pre peak music. Brightness is a characteristic of musical sound closely related to the amount of high-frequency content within that sound. It can be considered metaphorically in terms of sharpness and colorfulness of sound, rather than dullness and colorlessness. Fullness can be considered in terms of compact-scattered and full-empty dichotomies, whereas peak music may be perceptually seem more scattered and empty than pre peak music.

Quantitative analysis only yielded a single dimension on which pre peak and peak music differed. This dimension was not described within the qualitative analysis, and in that sense the quantitative analysis may be complementary to the effort of identifying features of musical stimuli that differ between peak and pre peak music. However, we expected but did not find the quantitative analysis to converge with the qualitative analysis. For instance, tempo was identified in qualitative ratings as a feature on which pre peak and peak music differ, but tempo was not found to significantly differ between pre peak and peak music in the quantitative analysis. Despite this lack of convergence, the qualitative and quantitative approaches yielded complementary knowledge, which supports the use of both methods in the current report.

### Toward a model of musical features that support mystical experience

We propose a conceptual model in which music expressing the characteristics of peak period music identified within this study, and the principal characteristics that differentiate peak and pre peak music (including qualitative features and the quantitative perceptual feature of brightness), support the occurrence of a mystical peak experience during acute psychedelic drug effects. Though this model has been developed based on psilocybin therapy, we believe this model should be extended to and tested with psychedelic psychotherapy that utilizes other psychedelic drugs. This model could be empirically tested using at least two methods.

The first method would be to select musical stimuli from a large corpus of composed and performed music that either expresses or does not express these characteristics. Large-scale music information retrieval databases exist (https://labrosa.ee.columbia.edu/millionsong/) that could support this effort. While a corpus may not exist at the present moment in the exact form that would support identification of music based on qualitative features that were identified in this report, such a corpus could reasonably be developed using large-scale survey methodology or crowd-sourcing methods. A straightforward experimental design could test the effectiveness of music that expressed the characteristics of peak period music (e.g., “target” peak music) against the effectiveness of music that did not express the characteristics of peak period music (e.g., “off-target” peak music) in occasioning peak psychedelic experiences, as measured using questionnaires that assess subjective experiences during psychedelic drug effects (e.g., the Mystical Experience Questionnaire, or MEQ30) (MacLean et al., 2012; Barrett et al., 2015), as well as long-term outcomes of psychedelic drug sessions (Griffiths et al., 2008, 2011).

A second method could be to compose, perform, and record novel music that either expresses or does not express the characteristics of peak period music, and subsequently test the effectiveness of these novel musical stimuli in supporting peak psychedelic experiences. Music composition based on observational methods has been a mainstay of film scoring for almost a century (Wierzbicki, 2009), and automated methods for music composition have been developed using MIR technologies (Cope, 2000, 2005; Lucas et al., 2016; Williams et al., 2017). The model developed from the current findings might be extendable into an instruction set for composers and/or a mathematical algorithm containing weighted stimulus features that could be used to either dynamically control sound generation, or to generate novel musical compositions. This method may yield clear and powerful insights into the value of these features of music in supporting peak psychedelic experience.

Individual, personal associations with music are known to evoke strong memories and emotions within music listeners, and these associations are by nature very idiosyncratic (Janata et al., 2007; Janata, 2009; Barrett et al., 2010; Barrett and Janata, 2016). Emotions and memories evoked by personally relevant music are extra-musical elements that would constitute noise in a model that sought to understand the relationship between psychological support during psychedelic drug effects and acoustic and musical features of stimuli presented during those effects. To the extent that there are features of music that are supportive of peak psychedelic experiences, and to the extent that idiosyncratic associations with previously heard music may be uncontrolled factors in a psychedelic therapy session, the availability of novel music that expresses optimally supportive musical features for psychedelic therapy sessions would be of great value. Musical preferences are well-known to influence emotional experience during music listening (Rawlings and Ciancarelli, 1997; Rentfrow and Gosling, 2003; Kreutz et al., 2007; Vuoskoski and Eerola, 2011), and to the extent that musical features could be identified that are supportive of peak psychedelic experience, this may facilitate composition of novel therapeutic music in different genres and toward different tastes, to further optimize music listening during psychedelic experiences.

## Limitations

The small sample size limits the generalizability of our findings. Also, given the anonymous nature of the survey, we cannot be sure that respondents are completely independent in terms of their histories and training in the use of psychedelics for research or therapy. To the degree that individual respondents do not have independence in their training or approach, this may artificially inflate the degree of agreement between stimuli that we have observed in peak period music. However, there were a range of composers genres, styles, and pieces of music that were recommended for the peak period, and from this we gain some confidence that responses were somewhat independent.

The extent to which respondents may be influenced by similar traditions regarding the role of music in supporting psychedelic therapy sessions is also an open question. While Bonny and Pahnke offer recommendations of specific stimuli to present during the peak period, they do not give guidance regarding the features of music that determined these selections. One musical selection identified in this survey for support during the peak and the pre peak periods of psychedelic experience (*Adagio for Strings* by Samuel Barber) and two musical selections identified for support during the pre peak period of psychedelic experience (*Nimrod* by Elgar, and Brahms' German Requiem) were suggested by Bonny and Pahnke (1972). We removed *Adagio for Strings* from analysis, as it was suggested by our participants for both peak and pre peak periods of music, and thus was non-specific to either peak or pre peak periods. *Nimrod* and Brahms' Requiem were entered into analysis, and though there were no qualitative features that were consistent across all pre-peak music, *Nimrod* and Brahms' Requiem contained qualitative features that were consistent with some of the pre peak period musical recommendations. It is not surprising that these selections were considered for use in psychedelic therapy sessions, given their notoriety as emotionally affective monuments of Classical music, however it is notable that only three of the total 51 unique musical recommendations in this survey were recommended by Bonny and Pahnke. Importantly, the current report identifies features of music, including features of musical selections recommended by Bonny & Pahnke, that may indeed provide guidance regarding music that supports peak experience.

There are many motivations for using both qualitative and quantitative methods to identify features of music that are descriptive of peak and pre peak period music. Qualitative methods are subjective by definition, and they can involve great effort and time in terms of both the time it takes for a human rater to listen to and individually rate multiple features of multiple pieces of music, and the time it takes to find consensus on features of music when those raters disagree. In contrast, quantitative methods such as the methods used within this report are more objective by nature, are automatic and computationally derived, and thus take advantage of modern computing technology to reduce the time and effort required to analyze music. However, there are many aspects of musical composition and performance that still elude quantitative analysis. For instance, it is particularly difficult for automated methods to analyze and accurately identify the orchestration of polyphonic music. While we hoped that qualitative and quantitative methods would converge, this did not occur clearly in our report. For example, tempo was found to differ between pre peak and peak music in the qualitative analysis within this report, however pre peak and peak music were not found to differ significantly in the quantitative dimension of tempo. This may be due to the small sample size (10 survey completers and a total of 44 musical stimuli for analysis), and this may also be a case where expert raters are more sensitive to an aspect of musical composition than an automated computational algorithm. While this makes a strong case for the consideration of the quantitative dimension of Brightness as being different between pre peak and peak music, this does not necessarily mean that pre peak and peak music should not differ on other quantitative measures. Future empirical analysis and quantitative analysis using a larger sample of stimuli is recommended, and if future investigations include a much larger corpus of musical stimuli, quantitative methods may be necessary.

The use of therapists and psilocybin guides with the current study, rather than patients or volunteers within psilocybin studies, may be viewed both as a strength and a limitation. In terms of strengths, use of responses in this survey from experienced therapists may produce more generalizable knowledge than can be gained from individual patients or study volunteers. Therapists whose data were included in the current survey had facilitated many tens to thousands of psilocybin sessions involving music, whereas the average individual patient or study volunteer will likely have limited average experience with psilocybin, relative to that of an experienced therapist. Conversely, it is possible that the therapists who responded to the currently reported survey had strong personal biases regarding the music that they found to be supportive during psilocybin sessions, and these biases may have strongly influenced their recommendations and sense of what is “optimal.” Moreover, the survey does not directly investigate what patients or study volunteers have experienced during psychedelic sessions that utilized the music recommended in the survey.

## Conclusion

The current paper is a tentative first step in an important area of future research into the optimization of music for the support of peak psychedelic experiences. Music found to be supportive of peak psychedelic experiences was characterized by regular, predictable, formulaic phrase structure and orchestration, a feeling of continuous movement and forward motion that slowly builds over time, and lower perceptual brightness when compared to pre peak music. The consistency in features of music found to be optimal for supporting peak psychedelic experiences suggests that there may be something about the features of this music that is optimal for the peak experience. That there is less consistency in pre peak stimuli recommendations suggests that either there is no single “optimal” stimulus or that the optimal is yet to be identified. The features identified in this report were established in the context of psilocybin therapy, and the generalizability of these features has yet to be established with other psychedelic drugs. These features, if empirically validated with psilocybin and investigated and validated with other psychedelic drugs, will provide critical guidance for the standardization and optimization of the selection of music to support clinical trials with psychedelic drugs.

## Author contributions

FB, HR, and RG designed the study. FB collected the data. DS, JB, and FB analyzed the data and interpreted the findings. FB and HR provided preliminary drafts of the manuscript, and all authors edited the manuscript.

### Conflict of interest statement

The authors declare that the research was conducted in the absence of any commercial or financial relationships that could be construed as a potential conflict of interest.
